# Prognostic Factors for Visual Postsurgical Outcome in Rhegmatogenous Retinal Detachment—A Systematic Review

**DOI:** 10.3390/jcm14062016

**Published:** 2025-03-16

**Authors:** George Chereji, Ovidiu Samoilă, Simona Delia Nicoară

**Affiliations:** 1 Doctoral School of Medicine, “Iuliu Hat,ieganu” University of Medicine and Pharmacy, 8, V. Babes, Str., 400012 Cluj-Napoca, Romania; simonanicoara1@gmail.com; 2 Department of Ophthalmology, “Iuliu Hat,ieganu” University of Medicine and Pharmacy, 8, V. Babes, Str., 400012 Cluj-Napoca, Romania; ciprian.samoila@umfcluj.ro; 3 Ophthalmology Clinic, Emergency County Hospital, 3–5 Clinicilor Str., 400006 Cluj-Napoca, Romania

**Keywords:** rhegmatogenous retinal detachment, prognostic factors, pars plana vitrectomy, functional outcome, systematic review

## Abstract

Background: Rhegmatogenous retinal detachment (RRD) is an ophthalmological emergency that can lead to vision loss if left untreated. Pars plana vitrectomy (PPV) is the preferred procedure for most complex RRD cases with a high success rate. However, certain parameters related to the patient, disease history, or ocular presentation may influence surgical outcomes. Methods: A systematic review of studies from 2010 to 2023 was conducted using PubMed/Medline (National Library of Medicine, Bethesda, MD, USA) and Scopus (Elsevier, Netherlands). The main objective of this review is to present the most significant data published in the scientific literature over the last 10 years, focusing on the latest implications of prognostic factors affecting the success of PPV in RRD. The search included terms such as “prognostic factors”, “visual outcome”, “functional outcome”, and “rhegmatogenous retinal detachment”. The database search returned 3489 studies. The included studies had to involve participants with RRD treated mainly by PPV, a minimum of 10 participants, and at least a 6-month follow-up period. Studies were excluded if they involved patients with previous PPV treatment or trauma. After reviewing their abstracts, titles, and applying the exclusion criteria, 19 articles were selected. Because it is an ample and interesting topic, many authors explored the connection between prognostic factors involved in the management of RRD and the final visual and functional outcomes. Methodological quality was assessed using PRISMA guidelines. Results: various factors have been studied, ranging from classic ophthalmological parameters, such as refractive error, axial length, lens status, visual acuity, duration of symptoms, description of the RRD, and retinal tears, to more complex findings on optical coherence tomography. Conclusions: The factors that significantly influenced postoperative prognosis in RRD included preoperative best-corrected visual acuity (BCVA), duration of symptoms, macular status (on/off), extent of retinal detachment, presence of macular hole, and proliferative vitreoretinopathy (PVR). Disruption of the ellipsoid zone (EZ), presence of epiretinal membrane (ERM), and lack of external limiting membrane (ELM) integrity were associated with poorer outcomes following RRD surgery.

## 1. Introduction

Rhegmatogenous retinal detachment (RRD) is the most common retinal emergency that requires early surgical intervention and results in permanent vision loss in the case of delayed treatment. RRD is characterized by a separation between the neurosensitive retina and the retinal pigment epithelium (RPE). The pathogenesis of RRD is a complex process resulting from genetic and age-related changes in vitreous anatomy and vitreoretinal adhesion. Persistent vitreoretinal adhesion can cause traction and the formation of retinal breaks, which separate the neuroretina from the underlying RPE due to fluid influx. The physical and metabolic properties of RPE and neurosensitive retina that maintain normal retinal adhesion are highly intricate and, once exceeded, lead to disruption of the photoreceptor layer and vision loss [1,2]. The annual incidence of RRD is 12.17 per 100,000 population worldwide, with the highest incidence in Europe at 14.52 per 100,000 [3].

The primary surgical treatment options for retinal detachment include scleral buckling (SB), pneumatic retinopexy (PR), and pars plana vitrectomy (PPV). SB and PR are considered for primary and simple retinal detachments, while PPV is an effective and widely accepted primary surgery treatment, particularly for pseudophakic patients and complex detachments, with a success rate of 70 to 90% in uncomplicated cases [4,5], and a final success rate up to 97%. PPV offers improved visibility of the retinal periphery and breaks identification, facilitating faster foveal reattachment [6,7]. However, postoperative functional outcomes are not always consistent with anatomical success.

Over the past decade, there has been an increased interest in the prognostic factors in RRD influencing the best-corrected visual acuity (BCVA) following surgery for RRD. Various prognostic factors have been identified, including symptom duration [8,9], preoperative visual acuity [10,11], extent of retinal detachment [12], lens status, refractive errors, high myopia [13], optical coherence tomography (OCT) biomarkers such as disruption of the external limiting membrane (ELM) and ellipsoid zone (EZ) [14,15], epiretinal membrane presence, cystoid macular edema, persistent subretinal fluid [16,17], and central foveal thickness [18,19,20,21,22,23]. These factors have been reported to be associated with different final functional outcomes.

This systematic review aims to provide a comprehensive perspective on the factors involved in managing RRD and to help vitreoretinal surgeons establish patient prognoses and expectations for visual acuity recovery post-surgery. Currently, there are only a few reviews in the scientific literature focusing on this topic, making this work a valuable addition to the field. Furthermore, the past decade has seen the introduction of smaller-gauge PPV (23 to 27G), which may influence some findings from previous studies. A new systematization could be significant for establishing a future paradigm in RRD treatment.

## 2. Materials and Methods

We aim to provide a review of the scientific literature in the modern era, between 2013 and 2023, focusing on prognostic factors in RRD. A comprehensive search was conducted in PubMed/Medline (National Library of Medicine, Bethesda, MD, USA) and Scopus (Elsevier, Amsterdam, The Netherlands). The search strategy included the following keywords: “prognostic factors” OR “visual outcome” OR “functional outcome” AND “rhegmatogenous retinal detachment”. Citations were tracked for all relevant studies and review articles, and a manual search was carried out to identify additional studies. The review was not registered in any systematic review database or registry. However, compliance with Preferred Reporting Items for Systematic Reviews and Meta-Analyses (PRISMA) guidelines was maintained throughout the review process to ensure transparency and standardized reporting. This review used data from studies published in public databases and did not use data at the individual level, so there was no need for approval from the institutional review board.

The included studies had to involve participants with RRD treated primarily by PPV, with a minimum of 10 participants, and a follow-up period of at least 6 months. Patients treated by scleral buckle surgery were also included in the review. We did not include patients with previous pars plana vitrectomy treatment or trauma. Our inclusion criteria did not impose restrictions based on gender, age, or demographics.

However, we limited our selection to completed and published English-language articles. Studies where our outcome of interest was not explored were excluded. To identify additional studies, we manually reviewed the reference lists of the selected articles and other related reviews.

All references retrieved from the searches were exported to the Zotero library, where duplicates were identified and removed. First, studies were screened by title and abstract, followed by a full-text review of all eligible studies from the initial screening. Titles and abstracts of all retrieved studies were independently reviewed by two reviewers to assess eligibility. Data extracted from each study included study type, author, publication year, study design, demographic characteristics of participants, type of surgery, endotamponade, macular status, number of eyes treated, follow-up, prognostic factors analyzed, and study outcome.

## 3. Results

**Figure 1:**
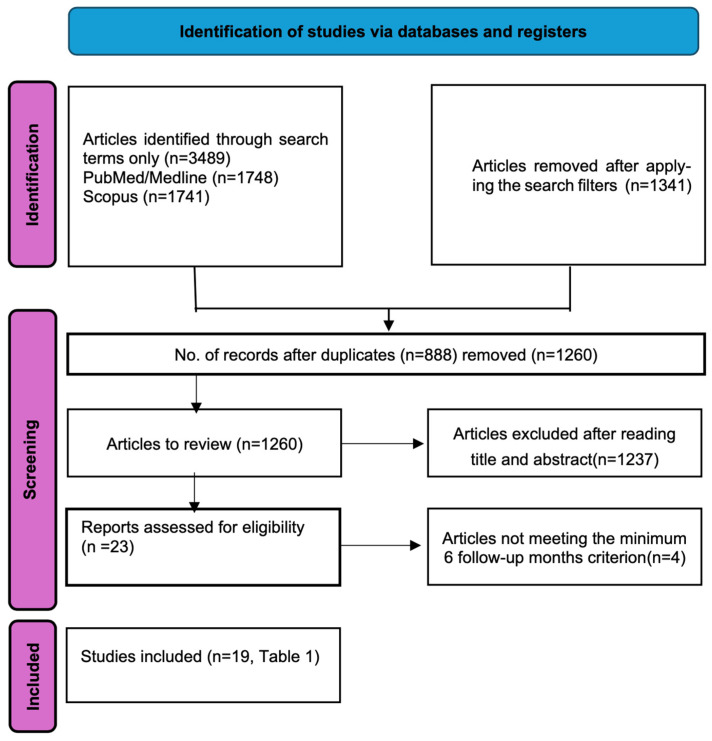
Flow chart of the selection of reviewed articles.

The database search identified 3489 articles with the keyword “prognostic factors”, “visual outcome”, “functional outcome”, and “rhegmatogenous retinal detachment”. After applying the filters (year: “2013 to 2023” and language: “English”) and removing duplicates, a total of 1260 articles remained. After reviewing their abstracts, titles, and applying the exclusion criteria (a minimum of 10 patients enrolled in the study, only RRD cases, and no previous PPV), 23 abstracts were selected. Finally, four articles were eliminated because they did not meet the minimum 6-month follow-up criteria (Figure 1).

Together, all the studies included 5232 patients with a mean age of 56.73 years, from which 1611 were male and 1015 were female. PPV was performed under either general or local anesthesia using 3-port, 23-, 25-, or 27-gauge instrumentation. At the end of the surgery, an air–gas mixture (sulfur hexafluoride-SF6 or perfluoropropane-C3F8) or silicon oil endotamponade was used. PPV was performed in 3450 cases, followed by 873 SB and 708 SB+PPV procedures (113 PR). The silicon oil was chosen as endotamponade in 376 eyes, gas mixtures in 1555 eyes, and air tamponade in 32 eyes. Not all studies provided demographic characteristics.

Table 1 illustrates a synthesis of the 19 studies included in the review, arranged in chronological order. Investigated prognostic factors, the number of follow-ups, and the main outcomes were also noted. Most studies reported changes in BCVA and OCT after the surgical treatment of RRD, which was influenced by several factors.

## 4. Discussion

Prognostic factors in RRD are numerous. Some of them are investigated in most of the studies selected here (BCVA at presentation, for example). Others (OCT biomarkers) are seen only in a few studies. However, knowing the prognostic factors is valuable for the discussion with the patient to anticipate the visual outcome. Furthermore, this knowledge could spark prevention programs for those factors that are modifiable (e.g., time from first symptoms to presentations) through screening and awareness campaigns in target populations.

The research groups of the selected 19 studies had different approaches and opinions on this topic.

### 4.1. Demographic Characteristics

A few studies reported that demographic characteristics (age, sex) were involved in the prognosis of RRD (Table 2) [26,33,38,39]. Chatziralli et al. [26] reported that increased age was an independent predictor of poor postoperative visual acuity in patients with RRD treated by PPV. The mean age was 66.2 ± 8.3 years and a 5-year increase in age meant a 4.11 letter decrease in vision (*p* < 0.001). This was previously reported in a study from 2017 on SB procedures [43]. Poulsen and associates [33] noted that the female gender was a strong predictor for poor visual outcomes and a higher reoperation rate in women than men (19.2% vs. 6.4%) was reported. In fact, in multivariable regression analysis, female gender (OR = 8.5 [95% CI 1.8–39.8]) was the strongest risk factor for poor visual outcomes. Age did not influence the final BCVA. The other studies included in the review did not observe any association of outcome and demographic factors (or these were not followed) (Table 2).

### 4.2. Best Visual Corrected Acuity

Preoperative BVCA has been previously reported to be an important prognostic factor for predicting postoperative BCVA [44,45,46].

Fourteen of nineteen studies included in our review reported a correlation between preoperative visual acuity and postoperative visual acuity. Benda et al. [25] reported that preoperative BCVA had the strongest statistical significance as a prognostic factor. A better visual acuity at presentation seems to achieve a better functional outcome after PPV, as Altindal et al. [27] showed. Poor BVCA at presentation (logMAR > 0.3) was an important predictor for poor final visual outcome, as noted by Poulsen et al. [33]. Baba et al. [38] reported that the probability of having a BVCA of logMAR 0.1 at 6 months postoperative was better in the eyes treated by PPV than SB (OR = 1.75, 95% CI 1.18 to 2.58; *p* = 0.005).

Table 2 presents the clinical studies that investigated BCVA as a predictive marker for final visual outcomes.

### 4.3. Duration of Symptoms

The duration of symptoms seems to be closely related to anatomical success and visual outcome. Most studies refer to the duration of symptoms as the time interval between the loss of central vision and the time of surgery. Studies [47,48] demonstrated that retinal detachment produced apoptosis in photoreceptor layers, visible within 24 h, peaking at 2–3 days, and dropping to a low level 7–28 days after retinal detachment. In Pastor et al. [8], a study from 2008, the mean duration of symptoms was 10.8 days in patients with a final VA < logMAR 0.3, 13.9 days in patients with a final VA between logMAR 0.4 and 0.7, and 27.9 days in those with a final VA higher than logMAR 0.7. The authors reported a significant correlation between the duration of symptoms and functional visual outcomes.

The crucial preoperative duration of symptoms seems to be 7 days; a duration longer than 1 week was found to be associated with poor postoperative visual outcome in the study of Chatziralli et al. [26]. Using a multivariate linear regression, Hirata et al. [32] reported that there is no significant correlation between the duration of symptoms and postoperative visual outcome. Patients who operated in less than 12 days had better VA (logMAR 0.2) compared to patients who operated on more than 12 days (logMAR 0.3) in the Baudin et al. [42] study. Additionally, they stated that the probability of having a final VA > logMAR 0.3 increased by 7% with each day from the first symptoms of visual loss to surgery, and no patient recovered more than logMAR 0.3 VA within 1 month of symptoms duration.

In the study of Guner et al. [28], postoperative BCVA was better at all follow-up examinations, 1, 6, and 12 months, in patients with prompt surgeries (less than 7 days post symptoms) versus delayed surgeries (more than 7 days post symptoms) (Table 3).

Sothivannan and associates [49] also concluded in their meta-analysis that the final BVCA, when macula-off RRD was treated in 0–3 days from symptoms onset, is better compared to its repair in 4–7 days, and visual outcome is better for macula-on treated in 0–24 h. Macula-off RRD repaired less than 3 days after onset of symptoms could have a logMAR 0.06 superior final visual acuity compared to 4–6-days delayed repair. Macula-on RRD repaired in less than 24 h could provide a logMAR 0.02 superior final visual acuity compared to more than 24 h delayed repair [48].

### 4.4. Macular Status

The status of the macula seems to play an important role in the prognosis of RRD. It has been highlighted in several studies that the destruction of photoreceptor layers occurs in eyes with macular detachment [50,51,52]. There are a few older studies that focus on prognostic factors in macula-on detachment. Among these, preoperative vision and single-surgery success have been associated with better visual outcomes [53,54,55,56].

Murtagh et al. [34] demonstrated that visual success (logMAR of 0.3 or less or an improvement of logMAR 0.3) was achieved in 431 (71.71%) of the eyes, from which 340 (78.89%) of the eyes were macula-on and 91 (21.11%) were macula-off detachments, emphasizing that macula-on detachment had a better anatomical and vision prognosis.

Table 4 synthesizes the association of BCVA and macular status in the studies of Altindal et al. [27] and Barequet et al. [29]. Both found that preoperative and postoperative BVCA were significantly better in eyes with macula-on retinal detachment [27]

### 4.5. Extent of Retinal Detachment

It has been reported that the extent of retinal detachment was associated with postoperative visual acuity [57,58]. Pollreisz et al. [59] observed that there was a correlation between the extent of retinal detachment and intravitreal proteins, such as IL-8 and TGFb-3, which were significantly increased in patients with more quadrants of detachment. These inflammatory changes could be the cause of retinal damage around detachment. Jung and Lee [60] presented a surgical success rate of 100% when the extent of retinal detachment was one quadrant and 54.8% when three or four quadrants were involved. Pastor et al. [8] noted a surgical success rate, defined as anatomical reattachment after 3 months of follow-up of 94.9% for one quadrant, 96.2% for two quadrants, 96.3% for three quadrants, and 84.6% for detachment of all quadrants.

Among the studies included in this review, Guner et al. [28] showed that patients with four quadrants of retinal detachment had significantly lower visual acuity at 12 months follow-up in comparison with patients with one quadrant detachment. Park et al. [31] also found a significant correlation between extensive retinal detachment and poor postoperative visual outcome. BCVA at 12 months follow-up after surgery was reduced by 0.133 for two-quadrant detachment and by 0.126 for three-quadrants detachment, in logMAR units. Gopal and associates [42] reported that patients with macula-on extending higher than 6 clock hours were significantly associated with worse logMAR final VA (mean logMAR 0.30) and were approximately three and a half times more likely to lose good vision than smaller retinal detachments. Sung et al. [39] noted that preoperative BVCA (logMAR) was 2.23 (±0.45) for patients with total RRD and 0.82 (±0.83) for partial RRD, and final BVCA (logMAR) was 1.88 (±0.83) for total RRD and 0.35 (±0.52) for partial RRD, with a success rate of 75% for total RRD and 96.6% for partial RRD.

### 4.6. Position of the Retinal Break

Breaks mostly occur in the superior retina. Geiger et al. [35] and Lee et al. [36] reported that more than half of retinal breaks were in the superior retina, more precisely 50.4% and 55%, respectively. Superior breaks seemed to have negative effects on postoperative BVCA in the study by Baba et al. [38].

Previous studies reported faster time to surgery for superior and temporal breaks compared to inferior and nasal breaks [36,61]. Superior RRD and posterior extension of SRF into the macula probably increase the risk of foveal detachment due to the gravity movement of subretinal fluid [61].

### 4.7. Macular Hole

The high prevalence of macular hole (MH) in macula-off retinal detachments, with a significant correlation to final visual acuity, was studied by Hostovsky and associates [40] on 44 patients. In their study, VA at presentation in patients with MH was logMAR 1.8667, versus logMAR 1.2993 in non-MH patients. The final postoperative VA was logMAR 0.90 in MH patients and logMAR 0.40 in non-MH patients. Sung et al. [39] reported that the macular hole was an important factor for the final best visual acuity, with a significant negative correlation.

### 4.8. Proliferative Vitreoretinopathy

Proliferative vitreoretinopathy is one of the most frequent causes of failure of RRD repair, with an incidence of 5–10% [62,63,64]. Chatziralli et al. [26] reported that PVR was a significant prognostic factor for poor postoperative BVCA (*p* < 0.001), a result similar to Park et al. [31] (*p* < 0.001) and Zgolli et al. [37] (*p* < 0.005). Guner et al. [28] concluded that patients with PVR had significantly poorer preoperative and postoperative BVCA. However, Benda et al. [25] and Gopal et al. [42] did not find a significant association between PVR and final BVCA, respectively, *p* = 0.667 and *p* = 0.92. Table 5 presents the studies that found (or did not) a statistical correlation between PVR and BCVA.

### 4.9. Other Factors

Sung et al. [39] reported that the success rate of surgery was significantly lower in pseudophakic eyes with an increased risk of redetachment of 3.6 times. Gopal et al. [42] reported that the preoperatory phakic eyes that remain phakic during follow-up were more exposed to loss of good vision compared to preoperatory pseudophakic eyes (*p* = 0.01) and preoperatory phakic eyes that underwent cataract surgery (*p* = 0.02). On the other hand, other studies did not find an association between lens status and final BCVA (Table 5).

Baba et al. [38] reported that eyes treated by PPV with an IOP < 10 mmHg and refractive error < −5 D had a poorer BVCA at 6 months follow-up, but there was no significant correlation between axial length and final BVCA. However, Karacolu et al. [24] did not find a significant correlation between IOP and final BVCA. Other studies did not investigate this issue (Table 5).

Polana and associates [33] reported that patients who received SO as endotamponament had significantly worse BVCA at the month 30 follow-up in comparison to patients who received gas (logMAR 0.19 [–0.20 to 1.18] vs. logMAR 0.08 [–0.20 to 1.66], *p* = 0.02). SO was associated with a poorer final BVCA by Baba et al. [38]. The poorer postoperative vision can be explained by the fact that silicone oil is used in more complex cases with a poor prognosis and by reducing VA during the time of tamponade.

### 4.10. OCT Biomarkers

Advancements in retinal imaging using OCT have shown viable causes for poor vision and functional outcomes in RRD surgery despite successful anatomical outcomes. OCT biomarkers analyzed by the studies included here were ellipsoid zone damage, presence of epiretinal membrane or cystoid macular edema, central retinal thickness, subretinal fluid, status of external limiting membrane, and photoreceptors layers. These markers were investigated in reattached retinas immediately after the successful surgery.

Table 6 presents the studies that investigated OCT biomarkers, marking those that concluded a significant predictive role (association), and those that did not find an association. Studies that did not investigate OCT biomarkers are also noted (not available column).

#### 4.10.1. Ellipsoid Zone

The disrupted ellipsoid zone was noted as the most regular lesion and was determined as a prognostic factor for postoperative visual outcomes in many previous studies [65,66,67,68]. Wakabayashi et al. [69], in their evaluation of microstructural changes in RRD successful anatomic repair, noted that 23 (43%) patients had disruption of the ellipsoid zone, of which 9 (39%) had disruption of ELM, and the ellipsoid zone was restored in 64% of eyes during follow-up.

Benda et al. [25] reported that discontinuity of the ellipsoid zone on OCT was the only postoperative OCT biomarker associated with a worse postoperative visual outcome. Chatziralli and associates [26] supported the idea that a disrupted ellipsoid zone was associated with poorer postoperative visual outcomes. Most of the studies that investigated preoperative EZ found a predictive factor for poor final BCVA when the ellipsoid zone is disrupted, however, Guner et al. [28] did not find a significant association between EZ and final BCVA.

#### 4.10.2. Epiretinal Membrane

Hostovsky et al. [40], in their study on 44 patients, found a significant correlation between the presence of ERM and final visual outcome. They also reported that in the group of patients with symptoms of less than a 2-week duration, 3% had ERM, compared to the group with symptoms of more than a 2-week duration, where 30% had ERM.

In the study of Baudin et al. [41] on 115 patients, the incidence of ERM significantly increased from 6.1% at 1 month to 15.6% at month 12, and they linked the development of ERM at month 3 with lower final visual acuity. They also reported that patients with disruption of ELM and EZ had a median logMAR 0.5 (59.5 ETDRS letters) postoperative VA at 12 months, compared to patients without disruption of ELM and EZ, who had a median logMAR 0.2 (76 ETDRS letters) postoperative VA, at the same follow-up.

Benda et al. [25] concluded that there was not a statistically significant correlation between ERM and final BVCA, although it was recorded in 53% of patients.

#### 4.10.3. External Limiting Membrane

The external limiting membrane, together with Muller cells, forms the connection points in the photoreceptors layer, and maintaining these connections is considered to accelerate photoreceptors healing. The duration and extent of macular detachment seem to affect ELM integrity [70,71].

Guner et al. [28] noted that 80% of patients had intact ELM at 12 months follow-up, a restoration that was achieved by most of the patients included in the study at 6 months after surgery. They concluded that preoperative ELM integrity and postoperative ELM restoration, especially within the first year, are the main predictors for final visual outcome. Park et al. [31] reported that disruption of baseline ELM integrity decreased BVCA by 0.163 logMAR. Karacolu et al. [24] and Benda et al. [25] reported no significant correlation between ELM integrity and final BVCA.

#### 4.10.4. Other OCT Biomarkers

The preoperative photoreceptor length, defined as the distance between ELM and the outer end of the outer segment of photoreceptors on OCT images, was studied by Hirata et al. [32]. They demonstrated that the preoperative length of photoreceptors was significantly correlated with postoperative photoreceptor length and the integrity of foveal ELM and EZ. Multivariate linear regression analysis revealed that preoperative photoreceptor length and preoperative BVCA were significant and independent predictive factors associated with the final visual outcome. The retinal detachment causes apoptosis of photoreceptors, which leads to a shortening of the outer segment of photoreceptors and a decrease in BVCA. Patients with severe retinal damage and apoptosis before surgery have shorter preoperative photoreceptor length and low integrity of the microstructure of photoreceptors, resulting in a poorer final BVCA [16,19,72].

Baudin et al. [41] reported that persistent cystoid macular edema at 1 year post-surgery was a prognostic factor for poorer visual recovery, with a median VA of logMAR 0.4 (63 EDTRS letters) in the case of CME versus the median VA of logMAR 0.2 (75 EDTRS letters) without CME.

The limitation of the studies analyzed is that they are mostly retrospective. Furthermore, we excluded articles that were not in English. Larger prospective studies should be encouraged.

## 5. Conclusions

According to many studies included in this review, the factors that significantly influenced the postoperative prognosis in RRD were preoperative BCVA, duration of symptoms, macular status (on/off), extent of retinal detachment, macular hole, and PVR.

Lens status, intraocular pressure, refractive error, axial length, and the type of endotamponade (gas or silicone oil) did not influence the postoperative outcome.

The studies that investigated OCT biomarkers showed that the disruption of EZ, presence of ERM, and lack of ELM integrity were associated with worse outcomes following RRD surgery. A few studies reported that shorter photoreceptor length and persistent cystoid macular edema at 1 year post-surgery were associated with worse outcomes.

In the clinical management of RRD, prognostic factors play a crucial role in determining the severity of the detachment, setting realistic expectations, guiding surgical decision-making, and understanding risk factors and recurrence. Prognostic factors such as preoperative visual acuity, duration of detachment, macular status, and the presence of complications can help estimate postoperative vision. For instance, if the detachment persisted for an extended period or if there is significant macular involvement, the likelihood of returning to baseline vision may be lower. Additionally, prognostic factors can assist in informing the patient about the recovery time and potential complications that may arise. By incorporating prognostic factors into the clinical management approach, surgical treatment can be personalized for each patient, including any additional procedures. The presence of specific prognostic factors, such as the extent of detachment, type, localization, and number of retinal breaks, may influence the choice of surgery (PPV, SB, or PR). In cases of significant PVR or complex detachment, the surgeon may consider additional procedures, such as silicone oil or membrane peeling. Preoperative counseling regarding these additional procedures can help patients manage their expectations for a more complex recovery and alleviate anxiety about the procedure. This fosters better outcomes, informed surgical decisions, and realistic expectations for visual recovery, enhancing patient satisfaction and results. Regular follow-up is critical for detecting any recurrence of RRD and complications, ensuring the best possible outcome for the patient.

## Figures and Tables

**Table 1:** Studies focusing on prognostic factors for rhegmatogenous retinal detachment surgery.

Year	Type of Study	Type of Surgery	Endo-tamponade	Macula Status	No. of Eyes Treated	Follow Up	Prognostic Factors Analysed	Correlation Between Prognostic Factors and Postoperative BVCA	Surgical Success of Initial Surgery
2017, Karacorlu et al. [24]	Retrospective	PPV 23G	SO, Gas	Macula-off	44	12–36 months	BVCA, age, sex, refractive error, IOP, lens status, type, number and size of tears, number of quadrants, status of macula, time between vision loss and surgery, and OCT parameters.	High correlation between a shorter period of symptoms and a better postoperative VA. Significant correlation between epiretinal membrane formation and postoperative VA.	The primary anatomical success rate after surgery was not mentioned.
2020, Benda et al. [25]	Retrospective	PPV 23G, 25G	SO, Gas (C3F8, SF6)	Macula-on/off	88	6 months	BVCA, age, axial length, duration of symptoms, lens status, macula status, PVR, OCT parameters (CRT, ERM, EZ, and CME), and type of surgery.	Significant statistical correlation between preoperative BVCA, duration of symptoms, integrity of EZ, and postoperative BVCA.	The primary anatomical success rate after surgery was 93%.
2021, Chatziralli et al. [26]	Prospective	PPV 23G	Gas (C3F8, SF6)	Macula-off	86	24 months	BVCA, age, sex, duration of symptoms, PVR, lens status, retinal breaks, OCT biomarkers (CRT, SRF, IRF, EZ, and ELM).	Statistical correlation between age, duration of the symptoms, PVR, CRT, and disruption of EZ and ELM and postoperative BVCA.	The primary anatomical success rate after surgery was not mentioned.
2022, Altindal et al. [27]	Retrospective	PPV 23G,25G	SO, Gas (C3F8, SF6)	Macula-on/off	41	6 months	BVCA, age, sex, duration of symptoms IOP, lens status, status of macula, topography of RD, retinal breaks (size, number, and location), and endotamponade: gas or SO, PVR, and OCT biomarkers (IS/OS junction, ERM).	Significant correlation between preoperative BVCA and postoperative BVCA. Better BVCA in patients with macula-on RRD.	The primary anatomical success rate after surgery was 100%.
2022, Guner et al. [28]	Retrospective	PPV 23G	SO, Gas (C3F8, SF6)	Macula-off	75	14–72 months	BVCA, age, sex, duration of symptoms and surgery, status of macula, extent of detachment, PVR, type of surgery, and OCT biomarkers (CMT, SRF, ELM, and EZ CIZ).	Significant correlation between the extent of the retinal detachment and postoperative BVCA. Statistical correlation between duration of symptoms, integrity of outer retina layers, and postoperative BVCA.	The primary anatomical success rate after surgery was not mentioned.
2023, Barequet et al. [29]	Retrospective	PPV 23G	SO, Gas(C3F8)	Macula-on/off	152	6 months	Age, sex, duration of symptoms, lens status, status of macula, preoperative BCVA, and OCT biomarkers (CST, SRF, IRF, RPE, and ERM).	Significant correlation between macula- off and a poor visual outcome. Correlation between a shorter duration of symptoms and a better postoperative BCVA.	The primary anatomical success rate after surgery was not mentioned.
2015, Kang et al. [30]	Retrospective	PPV (23G) + SB	SO,Gas(C3F8)	Macula-off	27	12 months	Age, sex, axial length, duration of symptoms, lens status, status of macula, presence of PVR, number, size of retinal breaks, preoperative BCVA, and OCT biomarkers (CST, SRF, IRF, RPE, ERM, CFT, and ELM).	Cystic cavities in INL or ONL do not have a significant impact on visual outcome. Significant correlation between integrity of ELM and the photoreceptors layers (postoperative) and visual outcome.	The primary anatomical success rate after surgery was not mentioned.
2018, Park et al. [31]	Retrospective	PPV + SB	Gas (C3F8, SF6)	Macula-off	180	12 months	Age, sex, BCVA, axial length, duration of symptoms, lens status, status of macula, presence of PVR, refractive error, extent of the detachment, and OCT biomarkers (CMT, SRF and ELM integrity).	Significant correlation between large detachment, macula-off duration more than 7 days and disruption of ELM, and a poor postoperative BCVA. Correlation between preoperative BVCA and the final postoperative BVCA.	The primary anatomical success rate after surgery was not mentioned.
2019, Hirata et al. [32]	Retrospective	PPV (23–25G) + SB	Gas (SF6)	Macula-off	69	12 months	Age, sex, axial length, presence of PVR duration of detachment, presence of cystoid cavity, and OCT biomarkers: length of photoreceptor, height of subretinal fluid integrity of foveal ELM, and EZ.	Lengths of preoperative photoreceptor and the preoperative BCVA were significant and independent factors associated with the postoperative BCVA.	The primary anatomical success rate after surgery was not mentioned.
2019, Poulsen et al. [33]	Prospective	PPV (25G) + SB	SO, Gas (SF6)	Macula-on/off	84	30 months	BVCA, age, sex, axial length, IOP, status of lens, presence of PVR, location of RD, size and number of retinal breaks, and type of tamponade.	Factors significantly associated with a poor final BCVA were poor baseline BCVA, female gender, retinal detachment >6 clock hours, and SO used as tamponade.	The primary anatomical success rate after surgery was not mentioned.
2019, Murtagh et al. [34]	Retrospective	PPV (20–23G) + SB	SO, Gas, (C3F8, SF6) Air	Macula-on/off	613	6 months	BVCA, age, sex, axial length, duration of symptoms, status of lens, macular status, location of RD, size and number of retinal breaks, type of tamponade, and type of procedure.	Correlation between macula-on and better final BVCA. Significant correlation between BVCA at presentation and final visual outcome.	The primary anatomical success rate after surgery was 88.58%.
2019, Geiger et al. [35]	Retrospective	PPV + SB	SO, Gas, (C3F8, SF6) Air	Macula-off	131	6 months	BVCA, age, sex, duration of symptoms, status of lens, macular status, location of RD, size and number of retinal breaks, type of tamponade, and type of procedure.	Significant correlation between BVCA at presentation and final visual outcome. A higher number of retinal breaks is associated with a poor final BCVA.	The primary anatomical success rate after surgery was 93%.
2019, Lee et al. [36]	Retrospective	PPV + SB + PR	No data	Macula-on	423	12 months	Lens status, duration of symptoms, number of quadrants involved, RRD posterior extent, RRD extent closest to the fovea, number of retinal breaks, and quadrants with retinal breaks.	This study identified two positive prognostic factors (preoperative BCVA and single-operation anatomic success). Three clinical factors correlated significantly with a shorter time from presentation to surgery (shorter symptom duration, superior RRD location, and extension of subretinal fluid into the macula).	The primary anatomical success rate after surgery was 81%.
2020, Zgolli et al. [37]	Prospective	PPV (23G) + SB	No data	Macula-off	90	6–12 months	BVCA, age, type, number, and size of tears, number of quadrants, status of macula, PVR, time between vision loss and	Statistical correlation between preoperative BVCA, PVR, number of quadrants detached, macula status, duration of symptoms and	The primary anatomical success rate after surgery was not mentioned.
2020, Baba et al. [38]	Prospective	PPV (23G,25G,27G) + SB	SO	Macula-on/off	2192	6 months	BVCA, time to surgery, IOP, refractive error, lens status, type, size, location of tears, macular status, choroidal detachment, PVR, and type of procedure.	correlation between multiple quadrants of detachment (especially superior breaks), macula-off, long time between first symptoms and surgery, and a poor postoperative BVCA.	The primary anatomical success rate after surgery was 91.8%.
2020, Sung et al. [39]	Retrospective	PPV (23G) + SB	SO, Gas (C3F8, SF6)	Macula-off	132	6 months	BVCA, age, sex, status of lens, IOP, axial length, type, location, and extent of retinal breaks.	Pseudophakic eyes and macular holes are prognostic factors for a poor visual outcome in total RRD.	The primary anatomical success rate after surgery was 96.6% for partial RRD and 75% for total RRD.
2021, Hostovsky et al. [40]	Retrospective	PPV + SB	No data	Macula-off	44	6 months	BVCA, lens status, IOP, location, extent and number of retinal breaks, duration of retinal detachment, and OCT biomarkers (RD height, epiretinal membrane, macular hole, and subretinal depositions).	Correlations between visual acuity at presentation, height of the detachment and duration of symptoms, and final postoperative BCVA. Statistically significant correlations between presence of macular hole or epiretinal membrane and a poorer final postoperative BCVA.	The primary anatomical success rate after surgery was 93%.
2021, Baudin et al. [41]	Prospective	PPV + SB	Gas	Macula-off	115	12 months	BVCA, age, sex, status of lens, duration of symptoms, IOP, axial length, type, location, extent of retinal breaks, type of intervention and tamponament, and OCT biomarkers (ELM, EZ, SRF, CME, and ERM).	Presence of ERM at 3 months, low BVCA at 3 months postoperative, and a longer time to surgery were the main predictors for worse final BCVA.	The primary anatomical success rate after surgery was 100%.
2022, Gopal et al. [42]	Retrospective	PPV + SB	SO, Gas (C3F8, SF6) Air	Macula-on	646	6 months	Age, sex, BVCA, status of lens, duration of symptoms, type, location, number of retinal breaks, and type of intervention and tamponament.	Prognostic factors associated with loss of vision were preoperative BCVA, lens status, extent of retinal detachment, and need for additional surgery.	The primary anatomical success rate after surgery was 88.7%.

**Table 2:** Demographic and visual markers in the clinical studies included in the review (marked with the reference index).

Prognostic Factor	Statistical Correlation Between Prognostic Factors and Postoperative BCVA	No Statistical Correlation Between Prognostic Factors and Postoperative BCVA	Not Included in Statistical Analysis
Age	[26,38,39]	[24,25,29,32,33,40,41,42]	[27,28,30,31,34,35,36,37]
Sex	[33]	[24,26,29,32,40,41]	[25,27,28,30,31,34,35,36,37,38,39,42]
BCVA at presentation	[25,27,29,31,32,33,35,36,37,38,39,40,41,42]	[24,28,30]	[26,34]

**Table 3:** BVCA (pre and postoperative), depending on the duration of symptoms in the Guner et al. study [28] (* *p* < 0.05, prompt versus delayed surgery).

Duration of Symptoms	Preoperative BVCA	Follow up 1 Month *	Follow up 6 Months *	Follow up 12 Months *
Duration of symptoms <7 days before surgery	logMAR 1.22 ± 1.2	logMAR 0.68 ± 0.5	logMAR 0.55 ± 0.5	logMAR 0.53 ± 0.5
Duration of symptoms ≥7 days before surgery	logMAR 1.50 ± 1.3	logMAR 0.91 ± 0.4	logMAR 0.87 ± 0.5	logMAR 0.81 ± 0.5

**Table 4:** BVCA with macular status.

Authors		Macula-On	Macula-Off	
Altindal et al. [27]	Preoperative BVCA	logMAR 0.55 (median 0.7; range, 0–1)	logMAR 1.73 (median 1.8; range, 0.52–2.10)	*p* < 0.001
	Postoperative BVCA	logMAR 0.08 (median 0; range, 0–0.4)	logMAR 0.48 (median 0.3; range, 0–2.10)	*p* = 0.002
Barequet et al. [29]	Preoperative BVCA	logMAR 0.19 ± 0.20	logMAR 1.32 ± 0.58	*p* < 0.001
	Postoperative BVCA	logMAR 0.24 ± 0.20	logMAR 0.54 ± 0.79	*p* < 0.001

**Table 5:** Other factors followed in clinical studies included in the review (marked with reference index).

	Statistical Correlation Between Prognostic Factors and Postoperative BCVA	No Statistical Correlation Between Prognostic Factors and Postoperative BCVA	Not Included in Statistical Analysis
PVR	[26,28,31,37,39]	[25,33,38,42]	[24,27,29,30,32,34,35,36,40,41]
Lens status	[39,42]	[24,25,26,31,33,34,35,37,40,41]	[27,28,29,30,32,36,38]
Intraocular pressure	[38]	[24]	[25,26,27,28,29,30,31,32,33,34,35,36,37,39,40,41,42]
Refractive error	[38]	[25,30,31,32,33,37,39,41]	[24,26,27,28,29,34,35,36,40,42]
Axial length	-	[25,30,31,32,37,38,39,41]	[24,26,27,28,29,33,34,35,36,40,42]
Type of endotamponament (gas-SO)	[31,33,38]	[26,35,39,42]	[24,25,27,28,29,30,32,34,36,37,40,41]

**Table 6:** OCT biomarkers followed in clinical studies included in the review (marked with reference index).

OCT Biomarker	Statistical Correlation Between OCT Biomarkers and Postoperative BCVA	No statistical Correlation Between OCT Biomarkers and Postoperative BCVA	Not Included in Statistical Analysis
Ellipsoid zone	[24,25,26,30,31,32,37]	[28]	[27,29,33,34,35,36,38,39,40,41,42]
Epiretinal membrane	[24,40,41]	[25]	[26,27,28,29,30,31,32,33,34,35,36,37,38,39,42]
External limiting membrane	[26,28,30,31,32,37]	[24,25]	[27,29,33,34,35,36,38,39,40,41,42]

## Data Availability

Not applicable.

## Abbreviations

BCVA	Best corrected visual acuity
IOP	Intraocular pressure
CRT	Central retinal thickness
ERM	Epiretinal membrane
EZ	Ellipsoid zone
CME	Cystoid macular edema
SRF	Subretinal fluid
IRF	Intraretinal fluid
ELM	External limiting membrane
CIZ	cone interdigitation zone
CST	Central sub-fluid thickness
RPE	Retinal pigment epithelium
CFT	Central foveal thickness
CMT	Central macular thickness
PVR	Proliferative vitreoretinopathy
RRD	Rhegmatogenous retinal detachment
PPV	Pars plana vitrectomy
SO	Silicon oil
